# Molecular Design and Synthesis of Novel Salicyl Glycoconjugates as Elicitors against Plant Diseases

**DOI:** 10.1371/journal.pone.0108338

**Published:** 2014-09-26

**Authors:** Zining Cui, Jun Ito, Hirofumi Dohi, Yoshimiki Amemiya, Yoshihiro Nishida

**Affiliations:** 1 Guangdong Province Key Laboratory of Microbial Signals and Disease Control, Department of Plant Pathology, College of Natural Resource and Environment, South China Agricultural University, Guangzhou, China; 2 Department of Nanobiology, Graduate School of Advanced Integration Science, Chiba University, Chiba, Japan; 3 Department of Environment Science for Bioproduction, Graduate School of Horticulture, Chiba University, Chiba, Japan; 4 State Key Laboratory for Biology of Plant Diseases and Insect Pests, Institute of Plant Protection, Chinese Academy of Agricultural Sciences, Beijing, China; Natural Resources Canada, Canada

## Abstract

A new series of salicyl glycoconjugates containing hydrazide and hydrazone moieties were designed and synthesized. The bioassay indicated that the novel compounds had no *in vitro* fungicidal activity but showed significant *in vivo* antifungal activity against the tested fungal pathogens. Some compounds even had superior activity than the commercial fungicides in greenhouse trial. The results of RT-PCR analysis showed that the designed salicyl glycoconjugates could induce the expression of *LOX1* and *Cs-AOS2,* which are the specific marker genes of jasmonate signaling pathway, to trigger the plant defense resistance.

## Introduction

In the past two decades, the goal of sustainable and green agriculture had been inspiring researchers to explore the feasibility of restricting toxic agrochemical usage to reduce their impact on environment and food chains. One of the alternatives, which had been studied intensively in recent years, was to make use of plant defense potentials. Induction of plant defense resistance in crops by chemical or biological elicitors had drawn increasing attentions and was considered as a prospective strategy for disease control [1], [2].

During the long process of co-evolution, plants had evolved lots of defense mechanisms to deal with pests and pathogens. Following plant-pathogen interaction, a number of plant defense responses could be induced (e.g., callus deposition, PR-protein accumulation, *et al*.) at the site of infection, and also in uninfected tissues, activated by signal molecules associated with defense responses, which resulted in increased resistance to subsequent infections. The systemic acquired resistance is a “whole-plant” defense response that occurred following an earlier localized exposure to a pathogen. Activation of systemic acquired resistance required the accumulation of endogenous salicylic acid [3]–[5]. Besides the salicylic acid dependent defense signaling pathway, the others had also been reported. For example, endogenous jasmonic acid and methyl jasmonate were also the potent signaling molecules which could induce a large set of defense responses [6]. Systemic acquired resistance possessed low specificity, was not easily overcome by new pathogens which emerged frequently.

Chemical elicitors are agrochemicals which do not show a direct effect on pathogens and lacked fungicidal activity themselves but induce defense mechanisms, which clearly distinguish them from conventional pesticides [7]. Some of these agrochemicals are known to have signaling functions *in planta*, such as benzothiadiazole [8]–[13], which is a functional analog of salicylic acid, while others may mimic the attack of a pathogen, such as harpin [14] or flagellin [15].

Saccharides are known as potent elicitors [16]. The fragments of chitin and chitosan, which act as elicitors in many plants, could induce the production of nitric oxide and hydrogen peroxide in some plant epidermal cells [17]–[20]. Even neutral saccharides, such as *β*-glucans derived from cellulose or laminarin [21], [22], are capable of enhancing plant resistance. The accumulation of phytoalexins could be induced by branched hexa (*β*-D-glucopyranosyl)-D-glucitols in soybean [23], [24]. Oligoglucans with polymerization between 8 and 17 could induce the chitinase activity in tobacco BY-2 suspension cells [25], [26]. The phenolic pathway could be rapidly induced by the mannose and glucose disaccharides in *Rubus* cells [27]. It is evident that saccharides have the ability to trigger defense responses in plants, enhance resistance toward infection, and even support plant growth [28], [29].

**Figure 1:**
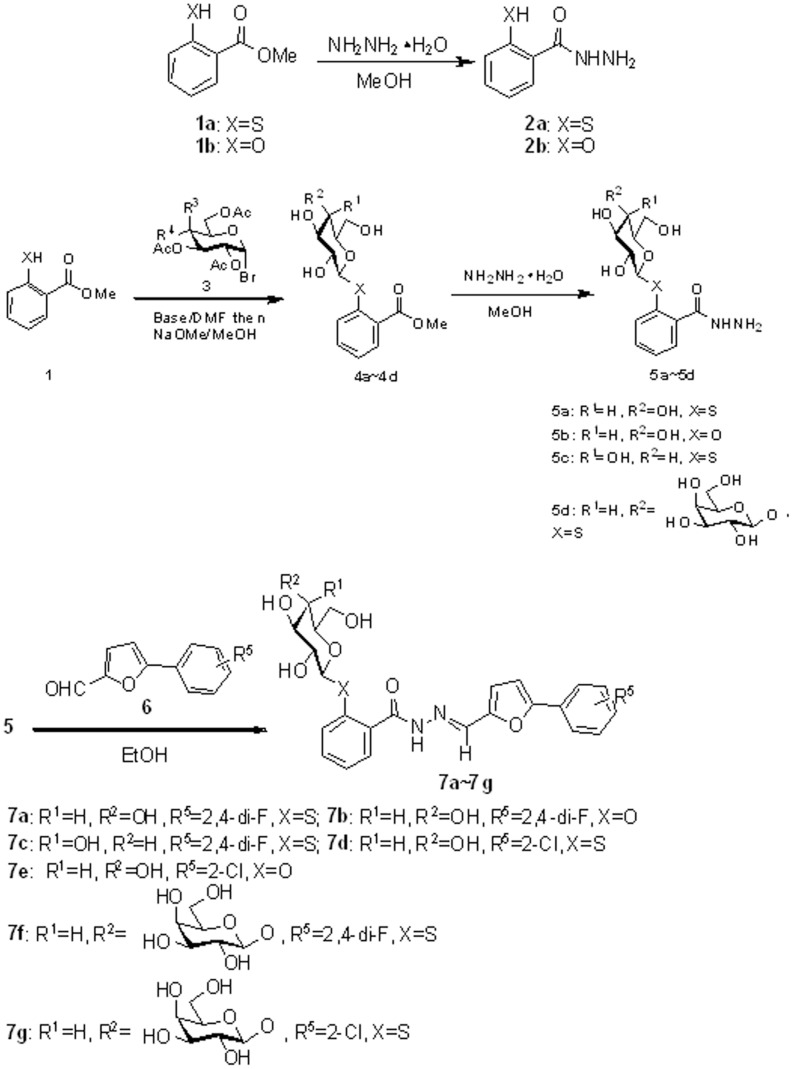
General synthetic procedure for salicylic glycoconjugates.

In our previous work, some 1,3,4-oxadiazole [30], benzoylureas [31]–[33], acylhydrazones [34], [35], diacylhydrazines [36]–[40], semicarbazide [41], pyrazole and 1,2,4-triazole [42] derivatives containing 5-phenyl-2-furan were designed and synthesized. All the compounds had considerable and diverse bioactivities such as insecticidal, fungicidal, and antitumor activities. Thus, 5-phenyl-2-furan was regarded as an active scaffold in drug design. In this study, we focused on the molecular design and synthesis of novel salicyl glycoconjugates as elicitors against plant diseases. We present here the preparation and characterization of the new elicitors based on salicylic acid and 5-phenyl-2-furan moiety (Figure 1), and show that these compounds could induce the systemic acquired resistance against pathogenic infections in cucumber.

## Materials and Methods

### Instruments

All the melting points were determined with a Cole-Parmer melting point apparatus (Cole-Parmer, Vernon Hills, Illinois, USA) while the thermometer was uncorrected. Optical rotation data were recorded on a KRUSS P8000 instrument (KRUSS, Karlsruhe, Germany). IR spectra were recorded on a Nicolet NEXUS-470 FTIR spectrometer (International Equipment Trading Ltd., Vernon Hills, Illinois, USA) with KBr pellets. ^1^H NMR spectra were recorded with Bruker DPX300 (Bruker, Fallanden, Switzerland) and JEOL JNM-ECS400 (JEOL Ltd., Tokyo, Japan), while tetramethylsilane was used as an internal standard. Analytical thin-layer chromatography was carried out on silica gel 60 F254 plates, and spots were visualized with ultraviolet light. Elemental analysis was carried out with a Flash EA 1112 elemental analyzer (Thermo Finnigan, Bremen, Germany). Mass spectra were measured on a Bruker APEX IV spectrometer (Bruker, Fallanden, Switzerland).

### Synthetic procedures

#### General synthetic procedure for hydrazides 2a and 2b

Preparation of hydrazides **2a** and **2b**: Esters **1a** and **1b** (30 mmol) was suspended in 100 mL methanol and reacted with 98% hydrazine monohydrate (60 mmol, 2.9 mL) under reflux for 12 h. The solid was filtered, washed with methanol and dried to afford hydrazides **2a** and **2b**.

*2-mercaptobenzohydrazide (* ***2a*** *).* Light yellow solid: yield 90%. m.p. 114–115°C. IR (KBr): *ν_max_* 3342, 3123, 1664, 1574, 1505, 1454, 1323, 1223, 1053 cm^−1^. ^1^H NMR (300 MHz, DMSO-*d_6_*): 4.65 (s, 2H, NH_2_), 5.16 (s, 1H, SH), 7.29–7.31 (m, 1H, PhH), 7.42–7.45 (m, 1H, PhH), 7.65–7.69 (m, 2H, PhH), 9.89 (s, 1H, CONH). ESI-MS: *m/e* 169.1 [M+H]^+^. Anal. Calcd. (%) for C_7_H_8_N_2_OS: C, 49.98; H, 4.79; N, 16.65. Found: C, 50.16; H, 4.91; N, 16.45.

*2-hydroxybenzohydrazide (* ***2b*** *).* White solid: yield 92%. m.p. 147–148°C. IR (KBr): *ν_max_* 3623, 3468, 1667, 1549, 1531, 1464, 1245, 1062 cm^−1^. ^1^H NMR (300 MHz, DMSO-*d_6_*): *δ* 4.53 (s, 2H, NH_2_), 5.23 (s, 1H, OH), 6.72–6.75 (m, 1H, PhH), 7.30–7.32 (m, 1H, PhH), 7.76–7.79 (m, 1H, PhH), 7.85–7.88 (m, 1H, PhH), 9.79 (s, 1H, CONH). ESI-MS: *m/e* 153.1 [M+H]^+^. Anal. Calcd. (%) for C_7_H_8_N_2_O_2_: C, 55.26; H, 5.30; N, 18.41. Found: C, 55.52; H, 5.14; N, 18.59.

#### General synthetic procedure for hydrazides 5a–d and hydrazones 7a–g

The key intermediates *hydrazides* ***5a–d*** were obtained almost quantitatively by the hydrazinolysis of compounds ***4a∼d*** in alcohol. Compounds ***5a∼d*** were condensed with 5-substituted phenyl-2-furfural to form the glycosyl hydrazones ***7a–g***. All the chemical characterization was given in reference [35].

### Bioassays

#### *In vitro* fungicidal activity

*In vitro* fungicidal activity of the salicylic glycoconjugates against *Colletotrichum orbiculare, Fusarium oxysporum, Rhizoctonia solanii,* and *Phytophthora capsici* were evaluated using mycelium growth rate test [43]–[45]. The tested compounds were dissolved in DMSO (dimethyl sulfoxide) and mixed with sterile molten potato dextrose agar to a final concentration of 50 µg/mL. *In vitro* fungicidal activity of the salicylic glycoconjugates against *Sphaerotheca fuliginea* was evaluated using colonized detached leaves method [43]–[45]. The conidial suspensions were prepared by seeding about 2×10^5^ spores mL^−1^ conidia in a 0.05% Tween 80 solution, and the DMSO solution of compounds (5000 µg/mL) was diluted with conidial suspension to a final concentration of 50 µg/mL. The solution was sprayed with a hand sprayer on the surface of the detached leaves which were inoculated with *S. fuliginea*.

*P. capsici* was maintained on oat medium at 17°C. *C. orbiculare, F. oxysporum,* and *R. solanii* were maintained on potato dextrose agar medium at 4°C. Five commercial fungicides: thiophanate-methyl, benomyl, chlorothalonil, validamycin, and dimethomorph were used as controls against the above mentioned fungal pathogens under the same conditions. Three replicates were performed. The relative inhibition rate of the synthetic compounds compared to blank control was calculated *via* the following equation:
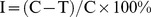



In which, I stands for the rate of inhibition (%), C is the diameter of mycelia in the blank control test (in mm), and T is the diameter of mycelia in the presence of tested compounds (in mm).

#### *In vivo* Antifungal Activity

Using the pot culture test [42], [46], the *in vivo* antifungal activities of the salicylic glycoconjugates against *C. orbiculare, F. oxysporum, S. fuliginea, R. solanii,* and *P. capsici* were evaluated in greenhouse along with five commercial fungicides, 70% thiophanate-methyl WP, 70% benomyl WP, 50% chlorothalonil WP, 3% validamycin AS, and 50% dimethomorph WP as controls.

The culture plates were cultivated at 24±1°C. Germination was conducted by soaking cucumber seeds in water for 2 h at 50°C and then keeping the seeds moist for 24 h at 28°C in an incubator. When the radicles were 0.5 cm, the seeds were grown in plastic pots containing a 1∶1 (v/v) mixture of vermiculite and peat. Cucumber plants used for inoculations were at the stage of two seed leaves. Ten plants were used for each treatment.

Tested compounds were confected to 2.5% EC (emulsifiable cocentration) formulations, in which pesticide emulsifier 500 (0.375%) and pesticide emulsifier 600 (2.125%) were the additives, DMSO (0.1%) was the solvent, and xylene was the co-solvent. The formulation was diluted to a concentration of 500 µg/mL with water. The solution was sprayed with a hand sprayer on the surface of seed leaves which were then inoculated with *C. orbiculare, S. fuliginea,* and *R. solanii*, respectively. Tested compounds and commercial fungicides were applied by irrigation at seedling stage, which were then inoculated with *F. oxysporum* and *P. capsici*, respectively. Three replicates for each treatment were applied.

Inoculations of *C. orbiculare* and *S. fuliginea* were carried out by spraying conidial suspension, and inoculation of *R. solanii* was carried out by spraying a mycelial suspension. *F. oxysporum* assay was carried out by embryo root inoculation, and *P. capsici* assay was carried out by irrigation inoculation.

Three replicates for each treatment were applied. After inoculation, the plants were maintained at 24±1°C and above 80% relative humidity.

The fungicidal activity was evaluated when the untreated cucumber plant (blank control) fully developed symptoms. The area of inoculated leaves covered by disease symptoms was assessed and compared to that of untreated ones to determine the average disease index. The relative control efficacy of compounds compared to the blank assay was calculated *via* the following equation:
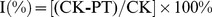
where I is relative control efficacy, CK is the average disease index during the blank assay and PT is the average disease index after treatment during testing.

#### RT-PCR for detection of pathogenesis-related gene expression

Tested compounds (500 µg/mL) were sprayed with a hand sprayer on the surface of the cucumber (*Cucumis sativus*) seed leaves, which were collected after 24 h, 48 h, and 72 h. The leaves were treated by liquid nitrogen. RNA isolation was performed with the RNAiso Plus Kit (Takara Bio). First-strand cDNA was synthesized from 100 µg/mL total RNA, which was quantified with QuantiT RNA Assay Kit (Invitrogen), by reverse transcription using the QuantiTect Reverse Transcription Kit (QIEGEN). Gene-specific primers (Table S1 in File S1) were designed and *actin* was used as the housekeeping gene [47], [48]. Each reaction mixture (30 µL) contained 1 µL of the cDNA template, 100 pmol of each primer, 10 µL of Premix Ex Taq HS (Takara Bio), and 20 µL reaction buffer. The thermal cycling conditions were as follows: initial denaturation (94°C, 5 min), followed by 40 cycles of denaturation (94°C, 30 s), annealing (30 s) and extension (72°C, 30 s), and one final cycle of extension (72°C, 5 min). Finally, RT-PCR products were separated by electrophoresis and visualized in 1% agarose gel.

### Ethics statement

No specific permits were required for the described field studies. No specific permissions were required for these locations. We confirm that the location is not privately-owned or protected in any way. We confirm that the field studies did not involve endangered or protected species.

## Results and Discussion

### Synthesis

The synthetic routes of 2-mercaptobenzohydrazide **2a**, 2-hydroxybenzohydrazide **2b** and glycosyl hydrazides **5a–d** were shown in Figure 1. The hydrazides **5a–d** were obtained almost quantitatively by hydrazinolysis of the esters **4a–d** in alcohol. Finally, the hydrazides **5a–d** were reacted with aldehyde **6** by condensation to form the glycosyl hydrazones **7a–g**.

### Fungicidal activity

**Figure 2:**
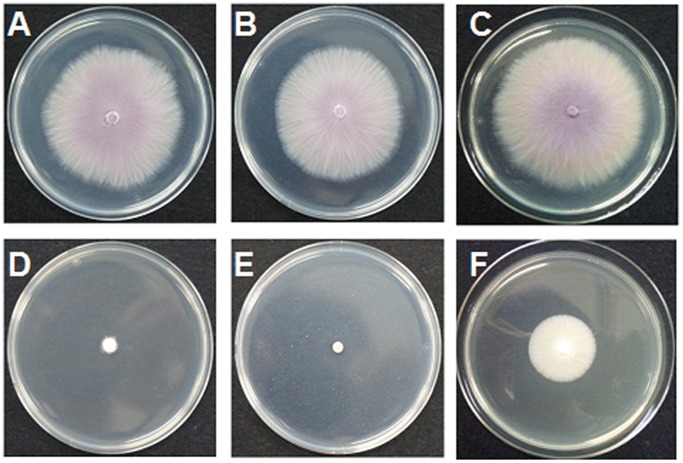
*In vitro* fungicidal activity against *Fusarium oxysporum*. A: blank control, B: **5d**, C: DMSO, D: **2b**, E: benomyl, F: **2a**.

**Figure 3:**
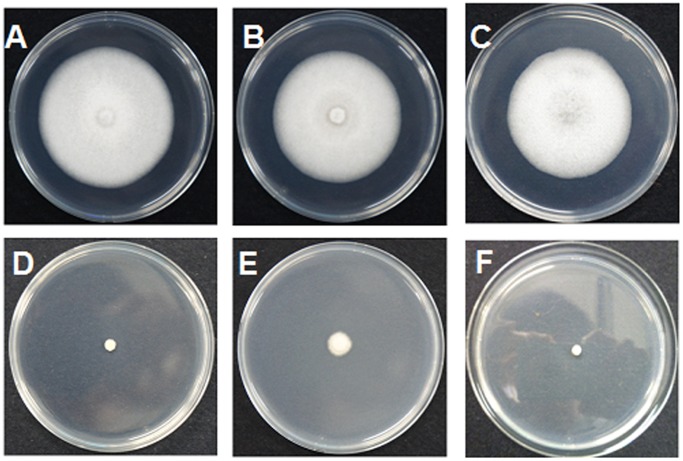
*In vitro* fungicidal activity against *Colletotrichum orbiculare*. A: blank control, B: **5d**, C: DMSO, D: **2b**, E: thiophanate-methyl, F: **2a**.

**Figure 4:**
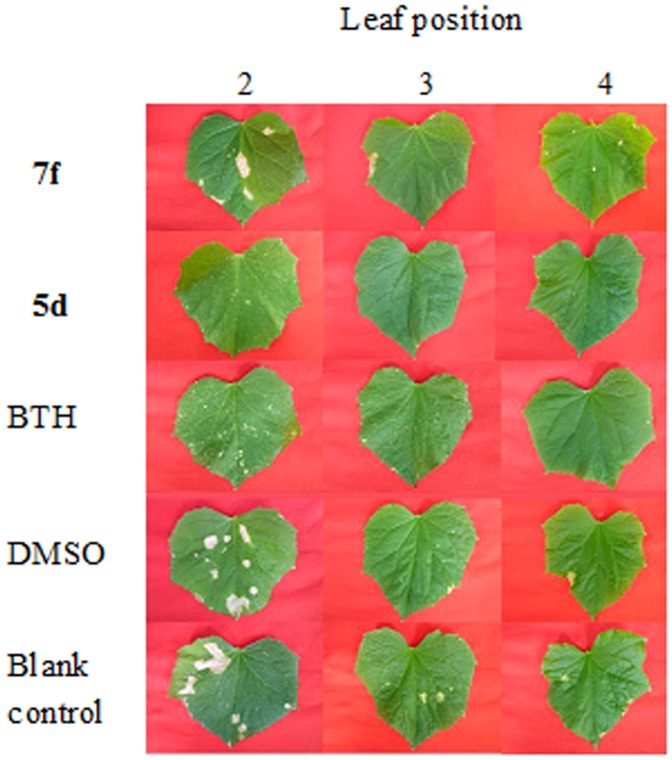
*In vivo* antifungal activity against *Colletotrichum orbiculare*.

**Figure 5:**
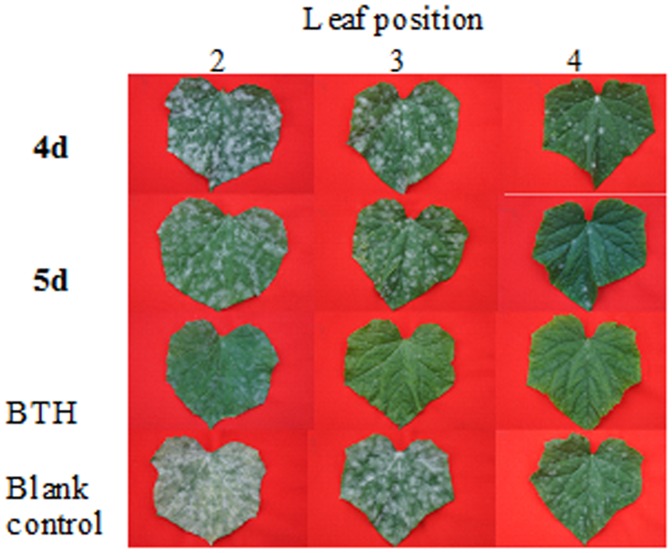
*In vivo* antifungal activity against *Sphaerotheca fuliginea*.

The *in vitro* fungicidal results were shown in Table 1. The hydrazides **2a** and **2b** showed excellent activity against the tested fungi (Figures 2 and 3). For example, the inhibitory rates of the hydrazides **2a** and **2b** against *C. orbiculare* were 97.3% and 95.4%, which were better than thiophanate-methyl (91.0%). After modification of sugars, the *in vitro* activity of all the derivatives was decreased and they exhibited poor inhibitory rates. Although the *in vitro* activity of these glycosides was not encouraging, the *in vivo* tests gave promising results (Table 2), with all the carbohydrate derivatives showing considerable activity, especially against *F. oxysporum* (Table 2), *C. orbiculare* (Figure 4), and *S. fuliginea* (Figure 5). Among them, hydrazide **5d** and hydrazone **7f** had activity of 71.0% and 74.9% on *F. oxysporum*, respectively, which is similar to the control benomyl (74.5%) against the same pathogen. **5d** also showed good activity of 68.6% and 73.9% against *C. orbiculare* and *S. fuliginea,* respectively. Some hydrazones **7** exhibited promising activity against *P. capsici*. For examples, **7c** showed an inhibitory rate of 83.5%, and the inhibitory rates of **7a**, **7d** and **7e** were more than 75%.

**Table 1:** *In vitro* fungicidal activity against five fungus species at 50 µg/mL.

Compd.	Inhibitory rate (%)				
	*C. orbiculare*	*F. oxysporum*	*S. fuliginea*	*R. solanii*	*P. capsici*
**2a**	97.3±2.0	73.0±2.2	73.1±2.1	86.5±2.3	56.2±2.2
**2b**	95.4±1.3	95.5±2.3	77.7±2.3	79.2±1.7	61.7±1.8
**4a**	11.3±1.0	12.2±1.1	28.1±2.0	27.9±1.6	13.2±1.1
**4b**	28.6±1.2	28.0±1.5	10.3±1.5	28.9±2.0	28.4±1.3
**4c**	12.9±1.2	9.3±0.4	6.5±0.6	18.1±2.0	12.9±1.7
**4d**	8.1±0.4	11.6±1.0	11.5±2.1	23.0±2.0	2.0±0.4
**5a**	15.2±0.9	7.4±0.2	10.4±1.7	26.1±1.0	21.6±1.0
**5b**	19.7±1.1	9.4±0.7	25.9±2.6	12.9±1.1	26.7±1.6
**5c**	2.2±0.3	3.2±0.1	1.3±0.8	16.3±2.4	23.8±1.2
**5d**	17.6±1.3	13.3±1.2	15.2±1.4	19.5±1.3	28.4±2.3
**7a**	24.4±1.2	35.3±1.7	28.5±2.0	15.4±1.0	21.4±1.3
**7b**	15.7±1.8	24.7±1.4	37.5±2.3	47.6±1.5	31.6±1.8
**7c**	25.5±1.7	33.3±1.6	17.5±1.1	39.4±2.0	38.6±1.2
**7d**	13.3±1.9	25.3±1.3	36.5±1.6	38.4±1.3	29.0±1.4
**7e**	25.5±1.0	21.5±1.1	19.6±1.7	19.8±1.0	41.5±1.5
**7f**	6.0±1.0	12.6±1.1	27.6±1.6	37.6±2.5	24.5±1.2
**7g**	11.3±0.6	8.2±0.8	12.4±1.6	21.5±1.5	15.7±1.1
DMSO	1.0±0.3	1.9±0.7	1.0±0.1	1.4±0.5	1.0±0.2
Fungicides^a^	91.0±1.3 a	98.2±1.2 b	97.5±2.1 c	91.0±2.1 d	91.2±2.5 e

a Control fungicides: a, thiophanate-methyl; b, benomyl; c, chlorothalonil; d, validamycin; e, dimethomorph.

**Table 2:** *In vivo* antifungal activity against five fungus species at 500 µg/mL.

Compd.	Inhibitory rate (%)				
	*C. orbiculare*	*F. oxysporum*	*S. fuliginea*	*R. solanii*	*P. capsici*
**2a**	51.8±2.0	55.2±2.2	48.1±3.1	51.7±2.2	33.2±1.3
**2b**	61.7±1.1	64.5±2.4	43.5±2.2	51.8±3.1	26.8±1.1
**4a**	45.6±2.6	43.6±2.3	41.9±1.1	49.7±2.3	12.9±1.2
**4b**	41.6±1.2	28.1±1.0	39.4±1.7	21.5±1.5	11.3±0.6
**4c**	51.6±1.7	59.7±2.1	49.3±2.3	17.4±1.9	3.2±0.9
**4d**	47.5±1.8	34.5±1.6	34.7±1.3	43.8±1.6	6.6±0.3
**5a**	50.3±1.3	54.5±1.2	50.8±1.7	26.0±1.3	40.4±2.2
**5b**	53.3±1.8	61.8±2.0	53.7±2.5	45.0±2.0	11.3±1.7
**5c**	34.6±1.6	55.6±0.8	54.5±1.3	34.5±1.4	24.2±1.0
**5d**	68.6±1.3	71.0±2.3	73.9±2.6	31.2±1.4	12.9±1.2
**7a**	41.3±0.5	53.8±1.5	52.3±1.1	45.5±2.1	76.0±2.2
**7b**	48.3±1.3	33.6±1.5	54.5±1.5	36.8±1.9	68.6±1.5
**7c**	54.8±1.9	62.6±1.6	34.5±0.7	28.6±1.5	83.5±1.3
**7d**	12.6±0.3	34.5±1.0	37.8±1.3	36.6±1.9	78.5±1.6
**7e**	54.3±2.5	54.6±0.9	23.3±1.2	33.2±1.8	77.5±2.0
**7f**	59.6±1.8	74.9±1.3	14.7±0.8	14.5±1.0	25.6±1.1
**7g**	54.8±1.5	40.3±1.2	42.8±1.3	33.9±1.5	34.7±1.0
DMSO	2.2±0.6	2.9±0.2	2.4±0.4	2.2±0.8	3.1±0.6
Fungicides^a^	76.8±2.3 a	74.5±2.3 b	94.6±1.7 c	81.0±2.7 d	91.2±2.4 e

a Control fungicides: a, 70% thiophanate-methyl WP; b, 70% benomyl WP; c, 50% chlorothalonil WP; d, 3% validamycin AS; e, 50% dimethomorph WP.

The bioassay results showed that the tested compounds had *in vivo* antifungal activity against pathogenic fungi of Ascomycota (*C. orbiculare, F. oxysporum* and *S. fuliginea*), Basidiomycota (*R.* *solanii*), and Oomycete (*P.* *capsici*). The observed *in vivo* antifungal activity also had some association with the issue of pathogen biology. The tested compounds exhibited activity not only against the obligatory parasite pathogen (*S. fuliginea*), but also against the facultative parasite pathogens (*C. orbiculare, F.* *oxysporum, R. solanii* and *P. capsici*). The tested compounds also showed good activity against the soil-borne fungal disease (*F.* *oxysporum, R. solanii* and *P. capsici*). Also, we confirmed that all of these test compounds were safe for the host plants.

### Defense activity of designed compound in plant

**Figure 6:**
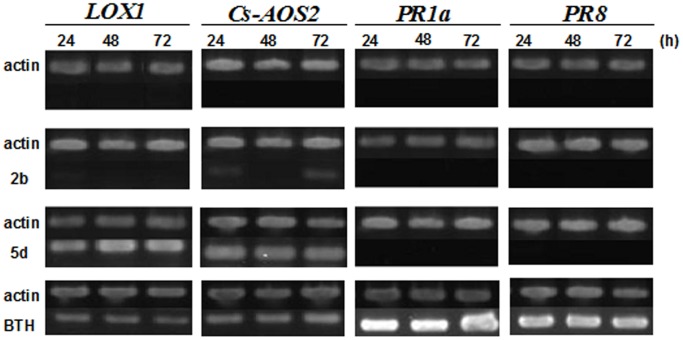
Effect of designed compounds on inducing the expression of pathogenesis-related genes in *Cucumis sativus*.

There are two important defense signaling pathways in plant system. One is mediated by salicylic acid and the other is mediated by jasmonic acid. In each defense pathway, there are specific marker genes which expression could be influenced by corresponding signaling molecules. In order to unveil the mode of action of our designed compounds, RT-PCR was performed to check the expression patterns of pathogenesis-related genes (*PR1a*, *PR8*, *LOX1*, *Cs-AOS2*) (Figure 6). Among them, *PR1a* and *PR8* were the specific marker genes mediated by salicylic acid, whereas *LOX1* and *Cs-AOS2* were the specific marker genes mediated by jasmonic acid. Our results showed that expressions of the *LOX1* and *Cs-AOS2* genes were significantly induced by hydrazide **5d**, and the expression level was comparable with that mediated by BTH (*S*-methyl benzo [1], [2], [3]thiadiazole-7-carbothioate). However, hydrazide **5d** had no obvious effect on the expressions of *PR1a* and *PR8*.

## Conclusions

A new series of glycosyl hydrazines and hydrozone derivatives were designed and synthesized. Their antifungal tests indicated that most of the salicylic glycoconjugates had no *in vitro* fungicidal activity but showed considerable *in vivo* antifungal activity. The plant defense activity showed that expressions of the *LOX1* and *Cs-AOS2* genes were significantly induced by hydrazide **5d**, but the compound had no effect on the expressions of *PR1a* and *PR8*. Intriguingly, although the designed compounds were the derivatives of salicylic acid, they did not mimic the mode of action of salicylic acid, but seem to follow the jasmonic acid mediated pathway to induce the plant defense resistance.

## Supporting Information

Table S1 Primers Used in This Study.
(DOC)
