# Supplementary material for: Molecular Design and Synthesis of Novel Salicyl Glycoconjugates as Elicitors against Plant Diseases
Source: PLoS One. 2014 Sep 26;9(9):e108338. doi: 10.1371/journal.pone.0108338 (PMC4178153; doi:10.1371/journal.pone.0108338)
Supplement: Table S1 — Primers Used in This Study. (DOC) [file pone.0108338.s001.doc]

**Molecular Design and Synthesis of Novel** **Salicyl Glycoconjugates as** **Elicitors against Plant Diseases**

Zining Cui^1,2,4,*^, Jun Ito^3^, Hirofumi Dohi^2^, Yoshimiki Amemiya^3^, Yoshihiro Nishida^2,*^

**1** Guangdong Province Key Laboratory of Microbial Signals and Disease Control, Department of Plant Pathology, College of Natural Resource and Environment, South China Agricultural University, Guangzhou 510642, China,

**2** Department of Nanobiology, Graduate School of Advanced Integration Science, Chiba University, 648 Matsudo, Chiba 271-8510, Japan,

**3** Department of Environment Science for Bioproduction, Graduate School of Horticulture, Chiba University, 648 Matsudo, Chiba 271-8510, Japan,

**4** State Key Laboratory for Biology of Plant Diseases and Insect Pests, Institute of Plant Protection, Chinese Academy of Agricultural Sciences, Beijing 100193, China.

**Page S2:** Table S1. Primers used in this study

*E-mail: [ziningcui@scau.edu.cn](mailto:ziningcui@scau.edu.cn) (ZC); YNishida@faculty.chiba-u.jp (YN).

Table S1. Primers used in this study

| Name | Sequence | Annealing temperature | The lengths of PCR products |
| --- | --- | --- | --- |
| *PR1a* | F：CTTCGTCGGTGTCCACAACG | 60 ℃ | 381 bp |
|  | R：AATTGCCACGGGGTTCGTAA |  |  |
| *PR8* | F：ATCGCCATCTATTGGGGTCA | 58 ℃ | 481 bp |
|  | R：TAGGTGAGCGTCTGGTATTG |  |  |
| *Lox1* | F：AAGGTTTGCCTGTCCCAAGA | 60 ℃ | 200 bp |
|  | R：TGAGTACTGGATTAACTCCAGCCAA |  |  |
| *Cs-AOS2* | F：GAAGAAAGACCTTGTGGTCG | 58 ℃ | 227 bp |
|  | R：ACACTGCTTGTTCTGAACCG |  |  |
| *actin* | F：TGGACTCTGGTGATGGTGTTA | 58 ℃ | 374 bp |
|  | R：CAATGAGGGATGGCTGGAAAA |  |  |
